# Coupling interval variability of premature ventricular contractions in patients with different underlying pathology: an insight into the arrhythmia mechanism

**DOI:** 10.1007/s10840-017-0309-8

**Published:** 2018-01-05

**Authors:** Lennart J. de Vries, Mihran Martirosyan, Ron T. van Domburg, Sip A. Wijchers, Tamas Géczy, Tamas Szili-Torok

**Affiliations:** 1 Department of Cardiology, Electrophysiology, Erasmus Medical Center, Rotterdam, The Netherlands; 2 Department of Epidemiology, Erasmus Medical Center, Rotterdam, The Netherlands; 3 Thoraxcenter, Department of Clinical Electrophysiology, Erasmus MC, ‘s-Gravendijkwal 230, Kamer BD416, Postbus 2040, 3000 CA Rotterdam, The Netherlands

**Keywords:** Coupling interval variability, Ventricular premature complexes, Idiopathic ventricular arrhythmia, Non-ischemic dilated cardiomyopathy, Familial cardiomyopathy, Arrhythmogenesis

## Abstract

**Purpose:**

Coupling interval (CI) variability of premature ventricular contractions (PVCs) is influenced by the underlying arrhythmia mechanism. The aim of this study was to compare CI variability of PVCs in different myocardial disease entities, in order to gain insight into their arrhythmia mechanism.

**Methods:**

Sixty-four patients with four underlying pathologies were included: idiopathic (*n* = 16), non-ischemic dilated cardiomyopathy (NIDCM) (*n* = 16), familial cardiomyopathy (PLN/LMNA) (*n* = 16), and post-MI (*n* = 16)-associated PVCs. The post-MI group was included as a reference, on account of its known re-entry mechanism. On Holter registrations, the first 20 CIs of the dominant PVC morphology were measured manually after which median ΔCI and mean SD of CI/√R-R (= CI of PVC corrected for underlying heart rate) were obtained. Two observers independently measured PVC CIs on pre-selected Holter registrations in order to determine inter- and intra-observer reliability.

**Results:**

The largest ΔCI was seen in the PLN/LMNA group (220 ms (120–295)), the lowest in the idiopathic group (120 ms (100–190)). The ΔCI in the PLN/LMNA group was significantly larger than the post-MI group (220 ms (120–295) vs 130 ms (105–155), *p* = 0.023). Mean SD of CI/√R-R in the PLN/LMNA group was also significantly higher than in the post-MI group (*p* = 0.044). Inter- and intra-observer reliability was good (ICC = 0.91 vs 0.86 and 0.96 vs 0.77, respectively).

**Conclusions:**

Low ΔCI and SD of CI/√R-R of idiopathic and NIDCM PVCs suggest that the underlying arrhythmia mechanisms might be re-entry or triggered activity. Abnormal automaticity or modulated parasystole are unlikely mechanisms. High CI variability in PLN/LMNA patients suggests that the re-entry and triggered activity are less likely mechanisms in this group.

**Electronic supplementary material:**

The online version of this article (10.1007/s10840-017-0309-8) contains supplementary material, which is available to authorized users.

## Introduction

Premature ventricular contractions (PVCs) are common both in patients with and without structural heart disease (SHD) [1]. Even in the population without apparent SHD, the incidence of PVCs is estimated to lie between 4 and 50% [2–5]. Although the independent prognostic importance of the PVC burden regarding adverse cardiac events (e.g., VT, sudden cardiac death, and heart failure) has not been clarified unambiguously, symptomatic PVCs can significantly reduce the quality of life (QoL) in both patient populations, and frequent PVCs can result in tachycardiomyopathy even in the absence of overt SHD [1, 6, 7]. It is therefore important to emphasize that the treatment of symptomatic and/or frequent PVCs can lead to a significant improvement of the QoL [6] and to the preservation/improvement of left ventricular function [7]. Catheter ablation (CA) has become a highly efficient alternative to medical therapy and is now in many cases being applied as a treatment of first choice [8]. However, a broad range of success rates have been reported in the literature, varying from 69% [9] to as high as 90% [8]. Incomplete understanding of the main underlying mechanisms of these arrhythmias may play a key role in this discrepancy.

One of the basic ECG characteristics of PVCs is the coupling interval (CI), which is defined as the distance between the onset of the preceding sinus QRS and that of the premature beat. An important feature of PVCs described in the literature is the variability of the CI. Although the determinants of CI variability and their clinical implications are not completely understood yet, early studies describe an association between higher CI variability and the incidence of VT and SHD among PVC patients [10, 11]. In addition, a relation between CI variability and the efficiency of anti-arrhythmic medical therapy has also been implicated [10]. Moreover, a recent publication has shown that CI variability might be able to discriminate between the precise anatomic origins of PVCs within the outflow tracts in patients with idiopathic ventricular arrhythmias (VAs) [12].

Although the variability of CIs is influenced by several factors (e.g., variation of the preceding cycle length, fluctuations in rhythmic distribution patterns, intermittent parasystole, and precipitancy of another ectopic source [13, 14]), their major determinant is believed to be the underlying arrhythmia mechanism. When PVCs have fixed CIs, then re-entry and triggered activity are among the most probable mechanisms. On the other hand, when PVCs exhibit variable CIs, then increased/abnormal automaticity or parasystole are more likely to be the source of rhythm disturbances [10, 15–17].

By describing the CI variability of PVCs in four distinct pathophysiological groups of myocardial disease, this study aims to shed more light on their underlying arrhythmia mechanisms. As the arrhythmogenic substrate for PVCs in patients with prior myocardial infarction (post-MI group) is well-described as being scar-related re-entry with a fixed CI (in cases of monomorphic PVC/VT,) this group of patients served as a control in our analyses. The arrhythmogenic substrates in structurally normal hearts and in non-ischemic myocardial disorders are less well-understood; therefore, we assessed the CI variability of PVCs in the following three groups: (i) patients with idiopathic VAs (idiopathic group), who exhibited PVCs in the absence of apparent SHD; (ii) patients with non-ischemic dilated cardiomyopathy (NIDCM group); and (iii) patients with familial dilated cardiomyopathy due to mutations in the genes encoding lamin A/C or phospholamban (PLN/LMNA group).

## Methods

### Patients

A database containing all performed CAs in our center was screened for patients undergoing VA ablation. Out of 345 VA ablations performed in our center between 2007 and 2015, 16 consecutive idiopathic VA patients, 16 NIDCM patients, and 16 post-MI patients were selected based on availability of Holter registrations. Sixteen PLN/LMNA cardiomyopathy patients from the inherited channelopathy and cardiomyopathy database were selected based on the same criteria. Selection of Holter registrations was based on the amount of PVCs recorded. A cutoff of 20 monomorphic PVCs of the dominant morphology was applied for selection, or otherwise the recording with the highest amount with a minimum of 4. All patient data was acquired from medical records by a trained physician. Pediatric patients were defined as younger than the age of 18 years. Arrhythmia origin was derived from electrophysiological studies, when available. We distinguished right ventricular outflow tract (RVOT), left ventricular outflow tract (LVOT), which includes coronary cusps and aortomitral continuity, and others (such as, ventricle walls, fascicular, or His region). Demographic data are presented in Table 1. Data collection was performed respecting the Health Insurance Portability and Accountability Act 1996.

**Table 1:** Patients demographics

	Post-MI	Idiopathic	NIDCM	PLN/LMNA	*p* value
Total Pts	16	16	16	16	
Age (years)	55 (53–63)	45 (40–61)	54 (38–60)	52 (40–57)	0.042
Pediatric (%/*n*)	0% (0)	0% (0)	6.2% (1)	6.2% (1)	0.559
Sex (%male/*n*)	93.8% (15)	50% (8)	56.2% (9)	50% (8)	0.028
Length (m)	1.78 ± 0.1	1.76 ± 0.1	1.76 ± 0.1	1.71 ± 0.1	0.296
Weight (kg)	88 ± 15	82 ± 15	83 ± 21	71 ± 14	0.029
BMI	28 ± 3	27 ± 3	27 ± 5	24 ± 3	0.026
Any anti-arrhythmic drugs	93.8% (15)	56.2% (9)	68.8% (11)	62.5% (10)	0.102
- Class I	0% (0)	12.5% (2)	0% (0)	0% (0)	0.103
- Beta blockers	81.2% (13)	37.5% (6)	43.8% (7)	56.2% (9)	0.064
- Class III	25.0% (4)	12.5% (2)	43.8% (7)	25.0% (4)	0.252
- Class IV	6.2% (1)	6.2% (1)	6.2% (1)	0.0% (0)	0.789
- Digoxin	6.2% (1)	0% (0)	6.2% (1)	37.5% (6)	0.006
LVEF					< 0.001
- Normal (> 55%)	6.2% (1)	93.8% (15)	18.8% (3)	31.2% (5)	
- Mild dysfunction (45–54%)	31.2% (5)	6.2% (1)	37.5% (6)	6.2% (1)	
- Moderate dysfunction (30–44%)	18.8% (3)	0% (0)	18.8% (3)	12.5% (2)	
- Severe dysfunction (< 30%)	43.8% (7)	0% (0)	25.0% (4)	50.0% (8)	
Monomorphic PVCs	13% (2)	100% (16)	44% (7)	6% (1)	< 0.001
Polymorphic PVCs	88% (14)	0% (0)	56% (9)	94% (15)	< 0.001
Ventricle of origin					< 0.001
- Left	100% (16)	25.0% (4)	50.0% (8)	n.a.*	
- Right	0% (0)	75.0% (12)	37.5% (6)	n.a.*	
- Both	0% (0)	0% (0)	12.5% (2)	n.a.*	
Arrhythmia focus				n.a.*	< 0.001
- RVOT	0% (0)	75.0% (12)	31.2% (5)	n.a.*	
- LVOT	0% (0)	25% (4)	25% (4)	n.a.*	
- Other	100% (16)	0% (0)	43.8% (7)	n.a.*	
PVC characteristics (of dominant morphology)					
- LBBB + superior axis	n.d.#	n.d.#	n.d.#	18.8% (3)	
- RBBB + superior axis	n.d.#	n.d.#	n.d.#	18.8% (3)	
- LBBB + inferior axis	n.d.#	n.d.#	n.d.#	25% (4)	
- RBBB + inferior axis	n.d.#	n.d.#	n.d.#	37.5% (6)	

Descriptive statistics are presented as mean ± SD for continuous variables, if normally distributed, or otherwise by median with (25th and 75th percentile). (*Not applicable: no ablation was done in this group of patients; therefore, the exact origin of the PVCs was not determined by electroanatomical mapping; PVC characteristics, indicative of PVC foci, are presented as a surrogate. #Not displayed: in case electroanatomical mapping is available, no PVC characteristics are displayed.)

*BMI* body mass index, *LBBB* left bundle branch block, *LVEF* left ventricular ejection fraction, *LVOT* left ventricular outflow tract, *RBBB* right bundle branch block, *RVOT* right ventricular outflow tract

### Measurement and determination of CIs

For every patient, PVC CIs were taken from a single 24-h Holter recording. Individual rhythm strips (depicting a certain time frame within the 24-h recording period, which usually encompassed approximately 10–60 s) were selected by designated Holter analyst, either manually or with the help of a computer software that generates an automatic event summary. These rhythm strips had been collected (and saved within the electronic documentation of each patient) based on their relevance with regard to the clinical inquiry posed by the referring physician. For the purpose of our analysis, we selected PVCs (and corresponding R-R intervals) without regard to the actual time periods these rhythm strips were depicting (throughout the 24 h registration period); thereby, ensuring that comparable numbers of day or nighttime registrations have been included. The first 20 PVC CIs of the dominant morphology were measured by hand with an accuracy of 20 ms. Additionally, the corresponding sinus R-R intervals preceding the selected PVC CIs were measured in order to correct the CIs for heart rate variability (as described below). The dominant PVC morphology was established by reviewing all the individual rhythm strips of the full 24-h Holter registrations. Distinct morphologies were then identified and grouped accordingly. Subsequently, the number of PVCs in each distinct group was determined, and the morphology, which belonged to the group with the highest PVC count (thus, the most frequently occurring morphology) on the analyzed rhythm strips, was considered the dominant morphology.

VTs were not included in this study. Two methods were used for assessing CI variability: (i) delta (Δ) CI (defined as the maximum minus the minimum CI duration) was defined for each patient, and the median and 25th and 75th percentiles of ΔCIs were presented for each group; (ii) the SD of CI/√R-R (the CI of each PVC corrected for the underlying heart rate) for each patient was defined, after which the SD of CI/√R-R per group was presented as mean with SD. The first step of the latter methodology was analogous to Bazett’s formula, which is used to correct the QT-interval by taking into consideration the underlying heart rate [18]. In our calculations of CI/√R-R, the R-R interval of the preceding sinus beat was used.

The monomorphic or polymorphic nature of PVCs and the amount of each morphology were determined subsequently (as described above). The monomorphic or polymorphic nature of PVCs was defined as possessing only one morphology or two or more morphologies on Holter, respectively.

### Inter- and intra-observer reliability

To determine inter- and intra-observer reliability, agreement and bias for CI measurements, the first two observers, both physicians, independently measured the PVC CIs on pre-selected Holter registrations from 32 patients (the idiopathic and NIDCM groups). When there was a discrepancy in the amount of CIs measured by the observers, this was discussed and a consensus decision was made. After a good reliability of the measurement method was established, one observer analyzed the two remaining groups.

### Statistics

The normality of distribution was assessed using the Shapiro-Wilk test. Descriptive statistics are presented as mean ± SD for continuous variables if normally distributed, or otherwise as median with 25th and 75th percentiles, where appropriate. Data were compared by one-way ANOVA or median test, as appropriate. The median test was used because equal variances between the groups were not assumed. Categorical data were expressed as percentages and compared with the chi-squared test. Intra-class correlation coefficients (ICC) were used to describe inter- and intra-observer reliability. Additionally, a Bland-Altman plot was used to assess the agreement between the two observers and to detect any bias. Statistical analysis was performed using SPSS version 21 (IBM Corp., Somers, NY). Statistical significance was defined as *p* < 0.05 (two-tailed).

## Results

### Patients and demographics

Patient demographics are presented in Table 1. Patients in the post-MI group contained more men (93.8%, *p* = 0.028), they were older (55 years (53–63), *p* = 0.042), and had a higher BMI (28 ± 3, *p* = 0.026). Digoxin was used more often in the PLN/LMNA group (37.5%, *p* = 0.006). Left ventricular ejection fraction (LVEF) was significantly different among the groups (*p* < 0.001): most patients (93.8%) in the idiopathic group had a normal LVEF and most patients (50%) in the PLN/LMNA group had severe LV dysfunction. All VAs in the post-MI group originated in the LV and none of them in the outflow tracts, whereas most of the VAs in the idiopathic group originated in the RV (75%), predominantly in the RVOT (75%). In the NIDCM group, the etiology was unknown (idiopathic) in 68.7% of the patients. The remaining etiologies included the following: SCN5A mutation, structural congenital heart defects, and limb-girdle muscular dystrophy.

### Coupling intervals

**Fig. 1:**
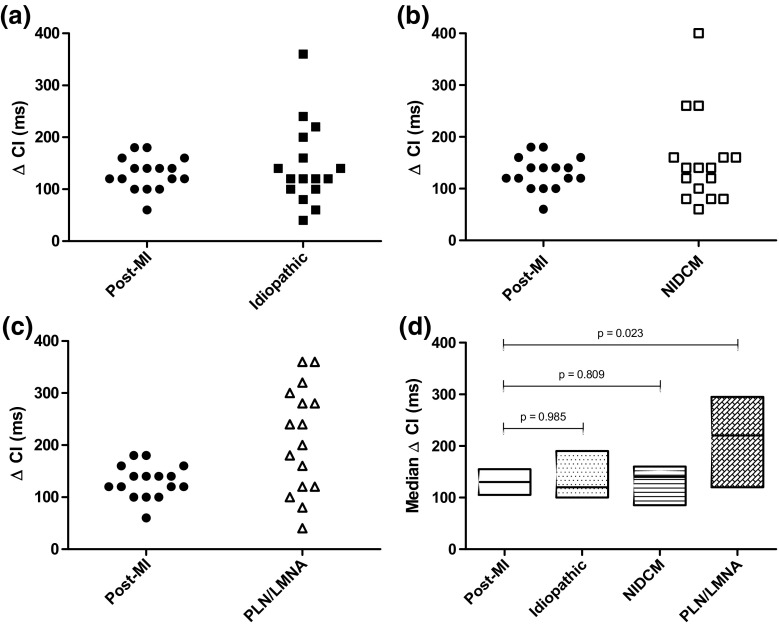
ΔCI compared to post-MI group. Median ΔCI per patient for post-MI group versus **a** idiopathic PVCs, **b** NIDCM PVCs, and **c** PLN/LMNA PVCs. The median ΔCI with 25th and 75th percentiles per group is shown in panel **d**

**Fig. 2:**
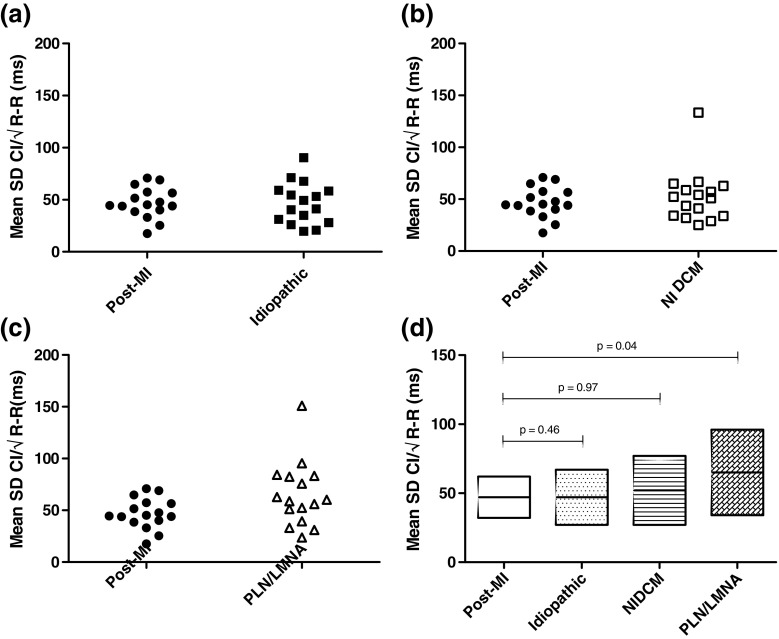
Mean SD of CI/√R-R compared to post-MI group. Mean SD of CI/√R-R per patient for post-MI group versus **a** idiopathic PVCs, **b** NIDCM PVCs, and **c** PLN/LMNA PVCs. The mean SD of CI/√R-R with standard deviation per group is shown in panel **d**

In four cases, there was a discrepancy in the amount of CIs measured by the observers, which was discussed followed by a consensus decision. Overall, the largest median ΔCI was seen in the PLN/LMNA group (220 ms (120–295)) and the lowest in the idiopathic group (120 ms (100–190)) (Fig. 1). The ΔCI in the PLN/LMNA group was significantly larger than in the post-MI group (220 ms (120–295) vs 130 ms (105–155), *p* = 0.023) (Fig. 1). The mean SD of CI/√R-R was as follows: post-MI 47 ± 15 ms; idiopathic, 47 ± 20 ms; NIDCM, 52 ± 25 ms; and PLN/LMNA, 65 ± 31 ms (Fig. 2). The mean SD of CI/√R-R in the PLN/LMNA group was significantly higher compared to the post-MI group (*p* = 0.044) (Fig. 2). The median amount of CIs measured was equal between the groups (*p* = 0.485). In the idiopathic group, there were no patients with polymorphic PVCs; in the PLN/LMNA, most patients (94%) had polymorphic PVCs (*p* < 0.001) (Table 1).

### Inter- and intra-observer reliability

The inter-observer reliability in a two-way mixed effect model was very good for the idiopathic group (ICC = 0.91) and good for the NIDCM group (ICC = 0.86) (Supplementary Fig. 1). The Bland-Altman plots for both groups show the observers were in good agreement regarding CI measurements (Supplementary Fig. 1).

The intra-observer reliability in a one-way random effect model was very good for the idiopathic group (ICC = 0.96) and good for the NIDCM group (ICC = 0.77) (Supplementary Fig. 2). The Bland-Altman plots for both groups show good agreement and no bias regarding CI measurements (Supplementary Fig. 2).

## Discussion

To the best of our knowledge, this is the first study in the literature that analyzes the CI variability of PVCs in several distinct subgroups of patients with VAs, in order to provide further insights into the underlying mechanisms of arrhythmogenesis related to different cardiac pathophysiology. The main findings of this study are the following: (1) although the underlying arrhythmia mechanisms might differ between the post-MI population and the idiopathic VA population (scar-related macro re-entry vs focal triggered activity), the CI variability of these groups were essentially identical, which indicates a similarly stable CI (fixed CI) for both re-entry and triggered activity within these pathophysiological subgroups. (2) The majority of the patients in the NIDCM group exhibited similar CI variability as the patients of the post-MI and idiopathic VA groups, which suggests that (despite a rather heterogeneous etiological background) the main mechanisms for arrhythmogenesis might essentially be similar to the ones of the previous groups, namely: scar-related micro/macro re-entry or focal-triggered activity with fixed CIs. (3) The patients of the familial dilated cardiomyopathy group (PLN/LMNA mutation group) exhibited high CI variability, which indicates that a mechanism different from re-entry or triggered activity might be responsible for PVC generation in this group. This mechanism may be abnormal automaticity, parasystole, or another more complex mechanism. Since a considerable portion of patients (10 out of 16) from this group were on either digoxin or class III anti-arrhythmic drug therapy, we additionally compared the CI variability of the subgroup of patients on these AADs with their counterparts not using these medications. We found no significant differences between the CI variability of these subgroups of patients (data not shown), implicating that although these AADs might be able to alter the PVC frequency, they might not have any effect on the underlying arrhythmia substrate (however, the numbers in each subgroup were considerably small with regard to statistical relevance; therefore, a firm conclusion from the results cannot be drawn).

### PVCs: General symptomatology and treatment

Symptomatic PVCs can present a considerable burden to patients, even with a structurally normal heart [6]. In addition to the significant impact of symptomatic PVCs on QoL, frequent PVCs can cause LV dysfunction, and in a minority of patients, they are also reported to initiate malignant VAs with a potential to cause sudden cardiac death. These outcomes should not be trivialized, especially when structural heart disease is present [7, 8, 19, 20].

Treatment of symptomatic and/or frequent PVCs can be challenging. More often than not, medical drug therapy is either inadequately effective, or its adverse side effects ensure that the cure becomes worse than the disease [8, 21]. Moreover, anti-arrhythmic drugs have not been demonstrated to reduce all-cause mortality in patients with or without structural heart disease [22]. On the other hand, although randomized trials of PVC suppression have not been performed, multiple studies indicate the high efficacy of PVC ablation [1]. In addition, technological advancements, such as magnetic navigation, have increased the safety of these procedures significantly [23]. Abolishment of frequent PVCs has been shown to reverse LV dysfunction in PVC-induced cardiomyopathy and improve QoL in patients with structurally normal heart [6, 7]. CA of some specific VA entities (such as idiopathic RVOT VAs or left posterior fascicular VAs) are reported to have very high success rates (> 95%) [8], and CA is increasingly being performed as a first choice therapy in these select cases. However, CA of certain other VA etiologies shows a much lower success rate in terms of arrhythmia termination [8]. The relatively wide range of success rates reported in the literature can at least be partially attributable to the different sites of VA origin [24] (i.e., technically challenging locations for the ablation procedure as, e.g., epicardial sites), and/or they might also be influenced by publication bias. On the other hand, incomplete understanding of the underlying arrhythmia mechanisms of VAs occurring in the presence of distinct cardiac diseases could represent another key contributing factor to the failure of CA procedures.

### Correlation of CI variability with arrhythmia mechanisms in different myocardial diseases

In order to further dissect the possible mechanisms that generate PVCs in different myocardial disease states, we analyzed the CI of PVCs in different patient populations. Although there is limited data available in the literature about the characteristics of CIs and their clinical significance, some reports suggest a connection between short CI duration (< 300 ms), a low prematurity index (< 0.73), and the potential of these parameters to indicate an increased risk for malignant VAs [25–27]. An earlier report of Komatsu et al. describes the variability of CIs as a characteristic that might have the potential to discriminate between groups of patients with low versus high risk for VT [10]. Additionally, their report suggests that higher CI variability has a tendency to occur in patients with organic heart disease, whereas patients with frequent PVCs in the absence of SHD tend to have a more fixed CI. Moreover, they also describe a correlation between CI variability and the efficacy of anti-arrhythmic drug therapy. Intriguingly, the characteristics of fixed and variable CIs that we describe in our study correspond well with the results of Komatsu et al., i.e., the mean SD of CI/√R-R of the post-MI group (47 ms), the idiopathic VA group (47 ms), and that of the NIDCM group (52 ms) all approach a range (35.4 ± 14.1 ms) that has been identified in their report as fixed CI, and the mean SD of CI/√R-R of the PLN/LMNA group (65 ms) fits well with the measures of their variable group (74.1 ± 28.6 ms).

In general, the following underlying mechanisms have been described in the literature to account for the generation of VAs: re-entry, abnormal automaticity, triggered activity, parasystole, and other more complex mechanisms involving such entities as, e.g., an arrhythmogenic milieu created by genetically defected ion channels and abnormal regulatory protein functions. Although it is not completely understood what determines the length and variability of CIs, and there is limited data on their association with the above mentioned basic arrhythmia mechanisms, it is generally presumed that re-entry and triggered activity have a rather fixed CI, whereas abnormal automaticity, parasystole, and other more complex mechanisms tend to result in CIs of higher variability [10, 15]. Hence, analyzing these interval changes might give us a good hint about the underlying mechanisms in different myocardial disorders.

From the four different disease entities included in our study, the arrhythmogenic substrate for VAs is best described and understood in post-MI patients. Unidirectional block and slow conduction in areas within myocardial scar tissue represent the pathological basis for the re-entry mechanism, which then gives rise to PVCs with a fixed CI (in case PVCs with the same morphology are taken into consideration, which of course represent the same underlying re-entry circuit with an identical exit site) [28]. Our results from the post-MI group indeed demonstrated low CI variability; hence, this group served as a control for the other three groups. One of them is the idiopathic VA group (patients with VAs in the absence of SHD). Most idiopathic VAs have their origin in one of the outflow tracts. Focal mechanisms have been described to account for this type of idiopathic VAs, which are usually localized in the RVOT (other less common sites include the LVOT and the aortic sinuses of Valsalva). Triggered activity secondary to cAMP-mediated delayed afterdepolarization is believed to be mainly responsible for this focal activity, but micro re-entry, abnormal automaticity, and modulated parasystole have also been implicated to account for this focal activity [29–33]. Our results showed a relatively low CI variability in this group (similar to post-MI patients), which in turn suggests that triggered activity and/or micro re-entry are the most likely mechanism for PVCs from the outflow tracts. However, as demonstrated by the three “outliers” in this group with a ΔCI above 200 ms (Fig. 1a), it is conceivable that in a small subset of patients different mechanisms might also play a role. A recent report of Bradfield et al. identified a subset of patients with outflow tract VAs, who exhibited more variable CIs than the majority of patients in this group. They postulated that the arrhythmia mechanism might be modulated parasystole in these patients and that the occurrence of this rather unusual mechanism might be related to the fact that the focal activity originates in more unique anatomic locations within the outflow tract (e.g., aortic sinus of Valsalva) [12]. However, we did not observe such a correlation, as all three patients exhibited PVCs with a common RVOT origin.

Since the patients in the NIDCM group represent a population with heterogeneous etiological backgrounds (in most cases, the underlying etiology remains unknown, other etiologies include valvular heart disease, hypertension, and sarcoidosis), a high CI variability would be expected in this group. Intriguingly, our data shows the opposite: PVCs with fixed CIs. In contrast to post-MI patients, the electrophysiological VA substrate in this group is not clearly defined. Although scar-related macro re-entry seems to account for the majority of monomorphic VTs, PVCs are believed to initiate primarily from the subendocardium by a focal mechanism without evidence of macro re-entry. The exact nature of the focal mechanism remained unknown so far, but our results might suggest that triggered activity and/or micro re-entry might be the most likely candidates. However, similarly to the previous subgroup of patients with idiopathic VAs, we identified several “outliers” in the NIDCM group ΔCI as well (see Fig. 1b), who exhibited higher CI variability, which could indicate the presence of different underlying arrhythmogenic substrates (abnormal automaticity or modulated parasystole). Correlations between the higher CI variability and clinical outcomes were beyond the scope of our present study.

The last group of patients in our present study was the group of familial dilated cardiomyopathy patients (PLN/LMNA group) who had a genetic disorder affecting the genes LMNA and PLN [34]. The LMNA gene encodes for two splice variants of proteins: lamin A and C that are members of the intermediate filament class of cytoskeletal proteins [35]. Phospholamban (gene product of PLN) is a calcium-regulating protein in the sarcoplasmic reticulum [36]. Intriguingly, we found that the CI of PVCs was highly variable in this group of patients, unlike that of the other three groups. This could suggest that common mechanisms such as re-entry and triggered activity are not likely to play a role in the genesis of VAs in this population. Other potential mechanisms could involve abnormal automaticity or modulated parasystole but more complex mechanisms cannot be excluded either. Especially if we consider that phospholamban plays an important role in intra-myocardial Ca^2+^-handling, it seems plausible that the gene alteration of such a regulatory protein might be able to create an arrhythmogenic milieu, which enables the generation of PVCs. How the altered intra-cellular ionic concentrations can specifically affect the mechanism of arrhythmogenesis and result in PVCs with variable CIs remains to be elucidated in future studies.

### Outcome implications and clinical significance

In an optimal case scenario, the treatment strategy of VAs should target the underlying arrhythmia mechanism. With CA, this mechanism can be targeted directly. For instance, in case of macro re-entry as the underlying mechanism (e.g., fascicular VAs), abolishment is accomplished simply by interrupting the re-entry circuit [37]. For VAs with a triggered activity-related mechanism (e.g., RVOT VAs), ablation of a focal target is required [38], as it is the case for automaticity. VAs precipitated by myocardial scar-related re-entry (e.g., post-MI VAs) should be targeted by substrate-based ablation [28]. Therefore, it is of importance that the underlying mechanism of the arrhythmia to be treated is clarified before deciding on a therapeutic strategy. Determination of CI variability could be a relatively easy and non-invasive method for aiding in the identification process. A better understanding of the arrhythmia mechanism could assist physicians in selecting optimal patient-tailored care and to determine the appropriate medical therapy. For instance, instead of beta-blockers, class III anti-arrhythmic drugs may be prescribed when the arrhythmia mechanism is found to be re-entry. Interestingly, ranolazine (originally intended as an anti-anginal drug) has recently been shown to reduce triggered PVCs based on its suppression of early or delayed afterdepolarizations [39, 40]. Additional studies are required to clarify whether this drug might be useful for the treatment of VAs for which the underlying mechanism is thought to be triggered activity.

### Limitations of the study

Although we tried to minimize any form of bias through our meticulous methodology, including (but not limited to) the assessment of inter- and intra-observer reliability of the measurement method, some limitations should be mentioned. Firstly, the use of Holter registrations with a registration speed of 25 mm/s for our CI measurements could introduce a minimal lack of precision. Secondly, the amount of PVC CIs that were counted per patient and the number of included patients were relatively small. The total patient count per group was limited by the amount of patients in the NIDCM group, upon which we matched the amount of included patients in the other groups. An automated CI measurement program counting PVC amounts of above 1000 per patient would be ideal. Additionally, inter- and intra-observer reliability was assessed with measurements from patients in the idiopathic and NIDCM group and not from patients in the two other groups. Finally, for patients from the PLN/LMNA group, EP studies were not available to confirm the clinical PVC origin or to invasively measure CIs. More basic studies are needed to clarify arrhythmia mechanisms, in order to improve our understanding of the different types of ventricular arrhythmias and to optimize their treatment strategies.

## Electronic supplementary material

ESM 1 (DOCX 200 kb)
