# Supplementary material for: Coupling interval variability of premature ventricular contractions in patients with different underlying pathology: an insight into the arrhythmia mechanism
Source: J Interv Card Electrophysiol. 2018 Jan 5;51(1):25–33. doi: 10.1007/s10840-017-0309-8 (PMC5797566; doi:10.1007/s10840-017-0309-8)
Supplement: Supplementary file 1 — (DOCX 200 kb) [file 10840_2017_309_MOESM1_ESM.docx]

**
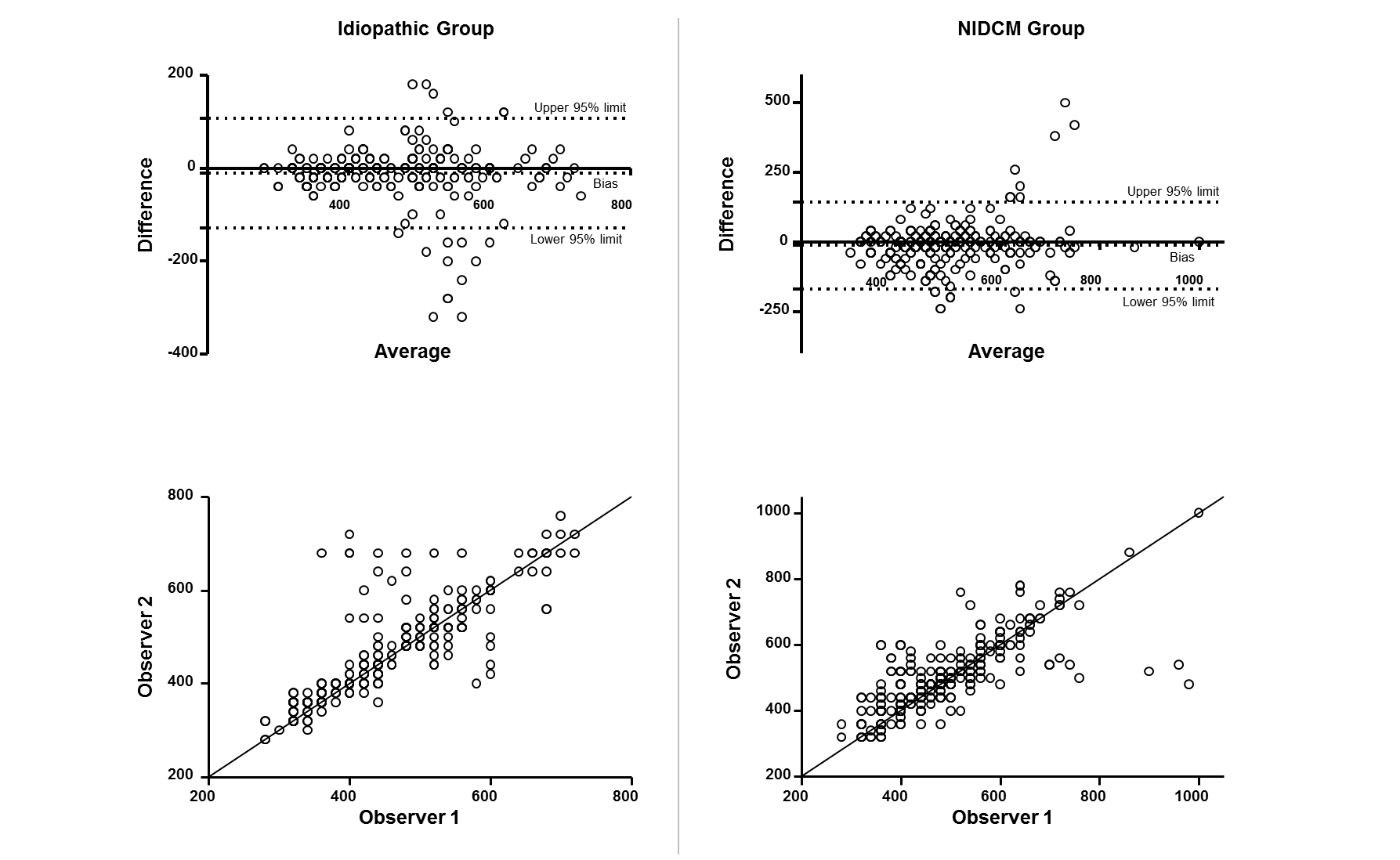
**

**Supplementary Figure 1. Bland-Altman plot and correlation for inter-observer reliability**

Demonstrating good agreement between the observers for both groups (ICC for idiopathic group = 0.91, ICC the NIDCM group = 0.86). The dotted lines in the Bland-Altman plot represent the upper and lower limits of agreement and the bias.


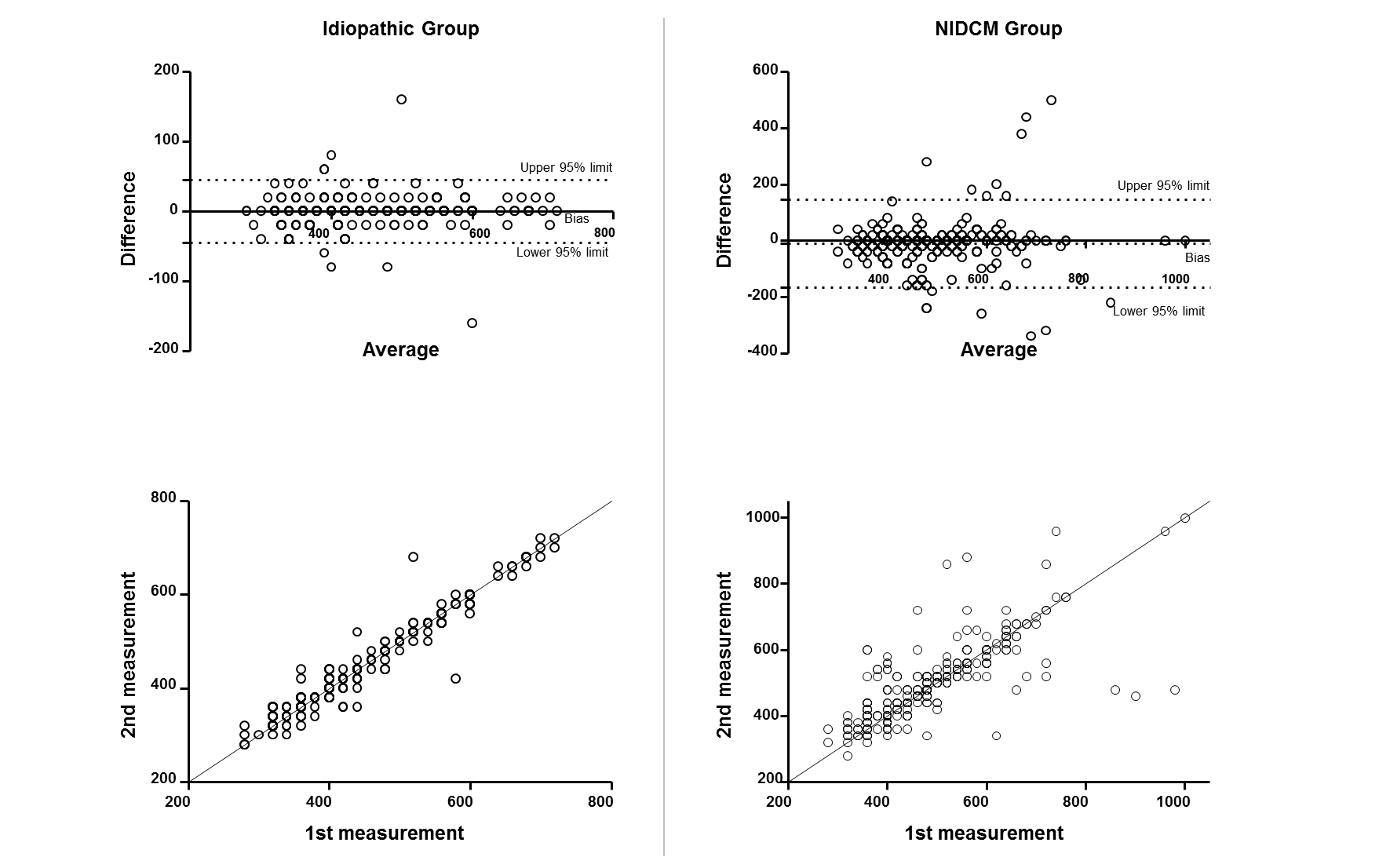


**Supplementary Figure 2. Bland-Altman plot and correlation for intra-observer reliability**

Demonstrating good agreement between the first and repeated measurements by observer 1 for both groups (ICC for idiopathic group = 0.91, ICC the NIDCM group = 0.86). The dotted lines in the Bland-Altman plot represent the upper and lower limits of agreement and the bias.
